# Mechanomyographic Measures of Muscle Contractile Properties are Influenced by Electrode Size and Stimulation Pulse Duration

**DOI:** 10.1038/s41598-020-65111-z

**Published:** 2020-05-18

**Authors:** Francisco Piqueras-Sanchiz, Saúl Martín-Rodríguez, Fernando Pareja-Blanco, Luis Baraja-Vegas, Jorge Blázquez-Fernández, Iker J. Bautista, Óscar García-García

**Affiliations:** 1 Physical Performance & Sports Research Center, Universidad Pablo de Olavide, Seville, Spain; 2 Department of Physical Education, University of Las Palmas de Gran Canaria, Las Palmas de Gran Canaria, Spain; 3 Department of Physiotherapy, Catholic University of Valencia, Valencia, Spain; 4 Faculty of Physiotherapy and Podology, Catholic University of Valencia, Valencia, Spain; 5 Laboratory of Sports Performance, Physical Condition and Wellness, Faculty of Education and Sport Sciences, Universidade de Vigo, Pontevedra, Spain

**Keywords:** Physiology, Biological techniques, Electrophysiology

## Abstract

The aim was to determine the effects of changing pulse duration and electrode size on muscle contractile properties. Thirty-six healthy young male participated in the study (age 24.8 ± 5.8 years; height 178.2 ± 0.6 cm; body mass 71.8 ± 7.3 kg; self-reported weekly moderate intensity activity 3.5 ± 1.2 h·week^−1^). Tensiomyography was used to assess rectus femoris (RF) and vastus medialis (VM) muscles neuromuscular properties of the dominant leg according to the electrode size (3.2–5 cm) and the stimulus length (0.2, 0.5, and 1 ms). Maximal radial displacement (Dm); Contraction time (Tc); Delay time (Td); Sustained time (Ts) and Half relaxation time (Tr) were measured. Relative and absolute reliability was quantified. To analyze the effects of the electrode and the stimulus length, a repeated-measures analysis of variance was used. Dm and Tc parameters showed for both muscles an excellent relative (0.95–0.99) and absolute reliability (1.6–4.2%). However, Ts and Tr showed low values of absolute reliability (4.4–40.9%). The duration of the stimulus length applied to the RF and VM and electrode size significantly influences muscle’s contractile properties (p < 0.05; η^2^_p_ = 0.09–0.60). The Dm increases substantially as the duration of the stimulus increases and with the use of the larger electrode in both muscles. However, Tc and Td are less affected by both conditions and not entirely clear. Practically, our study suggests that a stimulus pulse duration of 1 ms together with a 5 × 5 cm electrode is necessary to reach a reliable and reproducible assessment of both RF and VM muscles contractile properties.

## Introduction

Mechanomyography (MMG) is a set of different methods to record mechanical properties, such as muscle´s belly displacement and contraction time (Tc), in response to either voluntary or electrically stimulated muscle contraction^1^. Among the different MMG methods as vibromyography or sonomyography, tensiomyography (TMG) has received great attention in the last decade by the scientific and clinical community^2^. This method has been utilized in a variety of applications, including estimating myosin heavy chain composition^3^, determining muscle fiber type populations^4,5^, detecting stiffness or atrophy^6,7^, assessing athlete´s performance^8^, evaluating muscle fatigue in laboratory or field conditions^2^ or identifying muscle dysfunctions and treatment-effects^9,10^, among other functions.

TMG is one of the MMG techniques that records muscle contractile properties in response to electrically stimulated muscle contractions, which are evoked through the delivery of transcutaneous neuromuscular stimulations (TNS). Most of TMG protocols determine the maximal TNS with a ‘current ramp’ protocol. This methodology entails delivering a series of incremental TNS impulses by increasing amperage (mA) whilst keeping a constant voltage and stimulus length until a maximal muscle contraction is reached – as determined by maximal radial displacement (Dm). Little attention has received in the literature one of the aforementioned variables (i.e. stimulus length) of the TNS protocol. The stimulus length, i.e. the duration of the applied TNS stimulation, influences the magnitude of the electrical energy delivered to the muscle. Thus, stimulus length influences the amplitude and timing of the TMG waveforms. Unfortunately, a lack of a standardized protocol has led to a variety of TNS stimulus length being reported in humans ranging from 200 µs to 1000 µs in different muscles^11–13^. To our knowledge, one of the few studies that have addressed this question on MMG was published more than 10 years ago^14^. These authors tested a wide amount of stimulus length ranging from 50 to 500 µs, at 50-µs increments. They showed that the contractile properties of the muscle were considered stable at pulse durations above 300 µs, which was similar with previously published data for the same muscle^14^. Notably, these authors used a laser MMG sensor while TMG uses a contact-displacement sensor (CDS). In this regard, both laser and CDS offer good-to-excellent reliability, although significant systematic bias was identified with the CDS recording higher mean values^15^. However, it was previously identified that these differences may not be considered clinically significant. Despite the above, these authors also found that the wide limits of agreement (−19.0 ms and 25.2 ms) identified between half-relaxation time (Tr) (i.e., the time taken from 90 to 50% of Dm) measures, were considered unreliable from a clinical perspective^15^. This finding is consistent with data from a contemporary review on the reliability and measurement error of TMG, which concluded that Tr should not be used for clinical or research purposes^16^.

Inter-electrode distance (IED) for electrical stimulation is another key factor in the measurements of any MMG device. This parameter has been examined in some studies with several muscles, which have described that IED significantly influences TMG waveform, thus negatively affecting the measure^17–19^. Despite having studied IED little, the effect of the electrode size on the evoked response has been hardly studied in MMG devices, and only in the field of physical therapy to analyse the thresholds of sensory and motor excitation^20,21^. These studies observed that larger electrodes required greater voltage output but less pulse-charge density than the smaller electrodes. The above, transferred to TMG, means that electrode size could influence the TMG waveform, thus exhibiting different responses of the contractile properties depending on the size.

Therefore, it is important to increase the accuracy of TMG assessments while minimizing the measurement error to reach reliable data and compare between studies. It is also important for practitioners for the same issue, so they can be able to accurately and objectively compare several intra- and inter-subjects measurements over time.

We hypothesised that the larger the electrode size and stimulus pulse duration allowed by TMG, the greater the data accuracy and the less the measurement error. Therefore, the aims of this study were: 1) to determine the effect of changing the pulse duration on muscle contractile properties and 2) to discriminate if changing electrode size with different pulse duration affects muscle contractile properties.

## Methods

### Participants

Thirty-six healthy young and moderately active male volunteers (age 24.8 ± 5.8 years; height 178.2 ± 0.6 cm; body mass 71.8 ± 7.3 kg; self-reported weekly moderate intensity activity 3.5 ± 1.2 h·week^−1^) who had not suffered muscle or tendon injuries in the previous 6 months participated in the study.

The sample size was calculated for each evaluated muscle using the G*POWER software (Heinrich-Heine-Universität Düsseldorf. Germany). The results have showed that for the Dm of the rectus femoris (RF) and vastus medialis (VM), with an alpha of 0.05, a statistical power of 0.80 and an effect size of 0.25, at least 28 and 36 participants were needed respectively. Therefore, a sample size of 36 participants was selected.

### Compliance with ethical standards

All voluntary participants were informed of the research objectives and had the possibility to withdraw at any time from the investigation without any penalty. Informed consent was obtained from all individual participants included in the study. The study was conducted during 4 weeks, according to the Declaration of Helsinki, and the protocol was fully approved by the Ethics Committee of the Catholic University of Valencia (UCV2017-2018-73). The authors declare that they have no conflict of interest.

### Experimental design

A descriptive cross-sectional design was used in order to analyse the effect of the electrode size and the stimulus duration on the parameters obtained with TMG. All participants measurements were made on the same day, in the same room and under the same temperature and humidity conditions (23.3 ± 0.6 °C and 45 ± 6.6% respectively) measured with a weather station using an external sensor. The volunteers were instructed to refrain from moderate or heavy physical activities within 72 h prior the assessments.

Before the data collection, participants were familiar with the electrostimulation stimulus. A 10 min period was established lying face up on a stretcher in order to obtain a muscular relaxation state. All subjects were shaved and the evaluated muscle area was cleaned with alcohol to favour impedance. This was done both in the familiarisation and in the measurement period. New electrodes were used in each measurement using 3.2 or 5 cm self-adhesive circular electrodes. Different measurements were established in the VM and the RF muscles of the dominant leg according to the electrode size (3.2–5 cm) and the stimulus length (0.2, 0.5, and 1 ms), so that 36 measurements were obtained in each participant, 18 in the RF and 18 in the VM. The total duration of the evaluation was ± 60 min. The intervention conditions were randomised (see Table 1), that is, the muscle evaluation order, the used electrode sequence and the stimulus length was varied. i.e., subject 1 started in the VM with a 5 cm electrode and with 1 ms stimulus length, then 5 cm electrode being pulse duration 0.2 ms and finally electrode 5 cm and stimulus length 0.5 ms.

**Table 1:** Blocking subjects. A: 0.2 milliseconds (ms); B: 0.5 ms; C: 1 ms.

Electrode Size	3.2 cm			SUBJECT	5 cm		
SUBJECT	Blocking subjects				Blocking subjects		
1	A	B	C	1	A	B	C
2	A	C	B	2	A	C	B
3	B	A	C	3	B	A	C
4	B	C	A	4	B	C	A
5	C	A	B	5	C	A	B
6	C	B	A	6	C	B	A

### Contractile properties assessment

TMG was used to assess the neuromuscular properties of both RF and VM muscles of the dominant leg. Measurements were taken under static and relaxed conditions. Prior to performing the measurements, an accurate digital displacement-transducer (GK 40, Panoptik doo, Ljubljana, Slovenia) was perpendicularly positioned at the highest point of the muscle belly. The exact positioning of the electrodes for the RF and VM muscles were in concordance with the recommendations of the SENIAM project^22^. RF electrodes were placed at 50% on the line from the anterior superior iliac spine to the superior part of the patella and the VM electrodes were placed at 80% on the line between the anterior superior iliac spine and the joint space in front of the anterior border of the medial ligament. To assure the same electrode placement between the consecutive measurements, the point was marked with a dermatological pen. To elicit the twitch responses, two circular self-adhesive electrodes with difference sizes (3.2 or 5 cm) (Dura-Stick premium, CEFAR-COMPEX, Hannover, Germany) were connected to an electric stimulator (TMG-S1 doo, Ljubljana, Slovenia) and positioned on the muscle surface, following the arrangement of the fibres. Electrodes were placed symmetrically with a IED of approximately 5 cm, placing the positive electrode in the proximal area of the muscle above the measurement point and the negative electrode in the distal area below the measurement point, according to previous investigations^23^. Both RF and VM muscles were measured in the supine position, with the knee joint fixed at a 145° knee flexion angle, once again by means of a wedge cushion designed for such purpose. The electrical stimulation was applied with different duration of the stimulus (0.2, 0.5 or 1 ms) with a current amplitude of 100 mA (i.e., single-twitch). Single-twitch is defined as the contractile response to a single electrical impulse and it is a specific type of evoked muscle activity used to characterise the mechanical properties of a muscle or a single motor unit. Although to identify the maximal required stimulus amplitude, and thus the peak muscle response, a progressive incremental approach has been adopted in most studies on the topic^2,8^, using in some of them a single-twitch^24–26^. Typically, studies report that the peak response occurs at stimulus amplitudes between 60 and 100 mA, being in larges muscles, such as the lower limbs, much closer to 100 mA^2^. Therefore, the decision to use a single-twitch of 100 mA to homogenize the results derived from the different experimental configurations (i.e., electrode size and stimulation pulse duration). A 60 s rest period was allowed between each electrical stimulus to avoid fatigue or post-tetanic activation while a 120 s rest period was established between conditions. All measurements were taken by the same experienced evaluator.

In order to ensure the reliability of the TMG assessment, two measurements were taken in each participant in all conditions (stimulus length and electrode size). Between one and two minutes passed between the test-retest of each condition.

**Figure 1:**
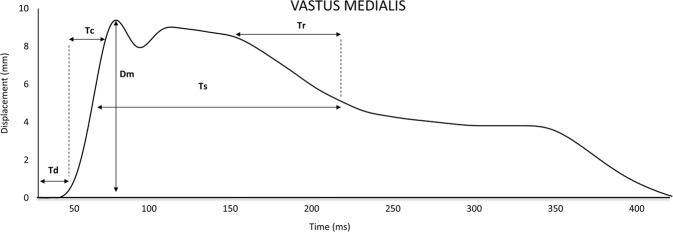
TMG parameters extracted of displacement-time curve of Vastus Medialis.

Each measurement involved recording the following parameters: maximal radial displacement (Dm; mm); contraction time (Tc; ms): Tc as the time from 10% to 90% of Dm; delay time (Td; ms) as the time from onset to 10% of Dm sides of the curve; sustained time (Ts; ms) as the time between 50% of Dm on both the ascending and descending sides of the curve; and Half relaxation time (Tr) was the time from 90% Dm to decline to 50% of the Dm in the relaxation phase^27^ (Fig. 1).

### Statistical analyses

Normal distribution of all variables was determined by Kolmogorov-Smirnov test, together with the Lilliefors test, in order to verify that the sample distribution was normal, linear and homoscedastic. Relative reliability was quantified by the intraclass correlation coefficient (ICC), along with a 95% confidence interval (CI), respect to the two measurements taken in each participant in all conditions. The ICC was calculated using a two-way-mixed effects and absolute agreement model^28^. ICC values under 0.50 were rated as low reliability, values between 0.50 and 0.75 indicates moderate reliability, values between 0.75 and 0.90 express good reliability and values greater than 0.90 indicates excellent reliability. Absolute reliability indices were expressed through the standard error of the mean (SEM, SEM%), minimum detectable change (MDC, %MDC) and coefficient of variation (CV) along with the respective 95% CI. The CV was calculated for raw data after being log-transformed^29^. A CV > 10% was interpreted as insufficient absolute reliability^17,30,31^. The SEM is an index for the precision of a measure and reflects the scores consistency within individual subjects^31^. The calculation of this index was made according Weir^32^ as follows equation: (SEM = √MS_E_), where MS_E_ is the mean square error term from the repeated measures ANOVA. The %SEM, which represents the relative amount of measurement error, was calculated according to Wagner *et al*.^33^ as follows equation: (SEM% = SEM/*M* × 100), being *M* the mean of the three TMG measurements. The MDC was calculated as equation: (SEM × 1.96 × √2)^32^. MDC% was expressed as equation: (MDC/*M* × 100)^30^, where *M* is the mean of the three TMG measurements. To analyse the effect of the electrode size (i.e., 3.2 vs. 5 cm) and the stimulus length (i.e., 0.2, 0.5 and 1 ms), an analysis of the variance of repeated measures (2 × 3 ANOVA) was used. Bonferroni post hoc test with adjustment for 95% confidence interval was used to compare the main effects and identify significant individual differences. The effect sizes in repeated measures ANOVA were reported as partial eta square (η^2^_p_) and interpreted as small (0.01), moderate (0.06), or large (0.14)^34^. An alpha level of p < 0.05 was considered statistically significant. All data were analysed using SPSS v24.0 for Windows (SPSS Inc., Chicago, IL, USA).

## Results

Dm and Tc parameters showed an excellent relative and absolute reliability with both electrode sizes and with all intensities for both muscles (Tables 2 and 3). Similarly, Td exhibited an excellent relative and absolute reliability in all conditions, except for the 3.2 cm electrode applied to the VM where the relative reliability was good. Ts showed a good to excellent relative reliability in both muscles, together with a good absolute reliability in the VM in all conditions. However, Ts exhibited an insufficient absolute reliability in the RF for all the conditions. Lastly, Tr showed insufficient absolute reliability for all the conditions for both muscles and a moderate to excellent relative reliability for both muscles. Based on the reliability results, Tr and Ts parameters were omitted in the ANOVA due to the insufficient absolute reliability.

**Table 2:** Reliability in tensiomyography parameters for Vastus Medialis muscle (n = 36).

VM	Electrode 3.2 cm																	
	M ± SD	Stimulus length 0.2					M ± SD	Stimulus length 0.5					M ± SD	Stimulus length 1				
		ICC CI 95%	CV%	SEM SEM%	MDC	MDC%		ICC CI 95%	CV%	SEM SEM%	MDC	MDC%		ICC CI 95%	CV%	SEM SEM%	MDC	MDC%
Dm (mm)	8.18 ± 1.50	0.97 (0.95-0.98)	3.0	0.24 ± 2.95	0.67	8.22	8.50 ± 1.51	0.97 (0.95-0.98)	3.2	0.26 ± 3.10	0.73	8.62	8.60 ± 1.36	0.97 (0.95-0.98)	3.1	0.23 ± 2.74	0.65	7.62
Tc (ms)	22.52 ± 2.10	0.95 (0.91-0.97)	2.2	0.49 ± 2.19	1.37	6.09	22.17 ± 2.04	0.95 (0.91-0.97)	2.3	0.47 ± 2.11	1.30	5.87	22.21 ± 1.94	0.95 (0.90-0.97)	2.2	0.43 ± 1.97	1.21	5.46
Td (ms)	22.38 ± 1.25	0.85 (0.75-0.91)	2.3	0.50 ± 2.23	1.39	6.21	22.23 ± 1.15	0.88 (0.81-0.94)	1.9	0.39 ± 1.77	1.09	4.92	22.24 ± 1.21	0.87 (0.79-0.93)	2.2	0.45 ± 2.02	1.25	5.62
Tr (ms)	67.10 ± 39.49	0.76 (0.62-0.86)	23.1	21.25 ± 31.66	58.92	87.80	75.95 ± 47.31	0.85 (0.75-0.91)	28.7	19.77 ± 26.03	54.81	72.16	71.09 ± 43.61	0.76 (0.62-0.86)	36.6	23.69 ± 33.32	65.66	92.37
Ts (ms)	168.60 ± 28.60	0.92 (0.87-0.96)	4.4	8.10 ± 4.80	22.46	13.32	180.5 ± 27.96	0.91 (0.86-0.95)	6.2	8.29 ± 4.59	23.00	12.74	175.6 ± 26.84	0.90 (0.83-0.94)	5.4	9.38 ± 5.34	26.01	14.81
	**Electrode 5 cm**																	
Dm (mm)	8.65 ± 1.34	0.97 (0.94-0.98)	3.0	0.25 ± 2.89	0.69	8.04	8.67 ± 1.36	0.96 (0.93-0.98)	2.9	0.31 ± 3.65	0.88	10.16	8.67 ± 1.30	0.95 (0.91-0.97)	3.8	0.26 ± 3.06	0.73	8.51
Tc (ms)	21.90 ± 1.98	0.97 (0.95-0.98)	1.6	0.33 ± 1.53	0.93	4.25	21.50 ± 1.97	0.95 (0.92-0.97)	2.0	0.43 ± 2.01	1.20	5.60	21.77 ± 2.01	0.95 (0.91-0.97)	2.4	0.47 ± 2.16	1.30	5.99
Td (ms)	22.21 ± 1.22	0.93 (0.88-0.96)	1.6	0.33 ± 1.49	0.92	4.15	22.08 ± 1.22	0.90 (0.83-0.94)	1.6	0.40 ± 1.84	1.12	5.11	22.06 ± 1.09	0.88 (0.81-0.93)	1.7	0.39 ± 1.78	1.09	4.96
Tr (ms)	81.93 ± 47.42	0.85 (0.76-0.91)	38.1	22.69 ± 27.69	62.90	76.77	75.37 ± 48.70	0.89 (0.81-0.94)	25.8	19.62 ± 26.03	54.38	72.15	62.76 ± 40.17	0.80 (0.68-0.88)	34.1	21.77 ± 34.68	60.34	96.15
Ts (ms)	175.41 ± 28.64	0.88 (0.81-0.93)	6.6	10.61 ± 6.04	29.42	16.77	182.65 ± 30.24	0.89 (0.81-0.94)	5.5	8.36 ± 4.58	23.19	12.69	182.58 ± 28.43	0.90 (0.84-0.95)	5.7	9.09 ± 4.98	25.20	13.80

Data are mean ± SD. VM: Vastus Medialis; Dm: displacement; Tc: contraction time; Td: delay time; Tr: half-relaxation time; Ts: sustain time; ms: milliseconds. CV: coefficient of variation; cm: centimeters; ICC: intraclass correlation coefficient; CI: confidence interval; SEM standard error of measurement; MDC: minimum detectable change.

**Table 3:** Reliability in tensiomyography parameters for Rectus Femoris muscle (n = 36).

RF	Electrode 3.2 cm																	
	M ± SD	Stimulus length 0.2					M ± SD	Stimulus length 0.5					M ± SD	Stimulus length 1				
		ICC CI 95%	CV%	SEM SEM%	MDC	MDC%		ICC CI 95%	CV%	SEM SEM%	MDC	MDC%		ICC CI 95%	CV%	SEM SEM%	MDC	MDC%
Dm (mm)	7.63 ± 2.10	0.98 (0.97-0.99)	4.1	0.28 ± 3.77	0.79	10.46	8.60 ± 2.51	0.98 (0.97-0.99)	3.7	0.31 ± 3.68	0.88	10.23	9.23 ± 2.19	0.98 (0.97-0.99)	2.9	0.28 ± 3.09	0.79	8.59
Tc (ms)	26.14 ± 4.79	0.98 (0.96-0.99)	3.0	0.67 ± 2.58	1.87	7.15	26.38 ± 4.77	0.98 (0.97-0.99)	2.4	0.57 ± 2.16	1.58	6.00	26.95 ± 4.67	0.98 (0.97-0.99)	2.4	0.57 ± 2.12	1.59	5.90
Td (ms)	24.50 ± 1.96	0.94 (0.90-0.97)	2.3	0.49 ± 2.01	1.35	5.58	24.82 ± 1.90	0.96 (0.93-0.98)	1.8	0.40 ± 1.61	1.08	4.46	24.95 ± 1.93	0.93 (0.88-0.96)	2.7	0.53 ± 2.14	1.48	5.96
Tr (ms)	73.77 ± 39.12	0.86 (0.77-0.92)	26.2	14.92 ± 20.23	41.37	56.09	65.21 ± 40.77	0.90 (0.84-0.95)	31.1	13.26 ± 20.34	36.77	56.38	73.52 ± 52.98	0.96 (0.92-0.97)	18.8	12.00 ± 16.32	33.26	45.24
Ts (ms)	117.77 ± 44.88	0.87 (0.78-0.92)	21.2	16.70 ± 14.18	46.29	39.35	105.07 ± 44.86	0.91 (0.86-0.95)	18.2	13.66 ± 13.00	37.87	36.04	109.30 ± 52.25	0.96 (0.92-0.97)	12.7	12.54 ± 11.47	34.77	31.79
	**Electrode 5 cm**																	
Dm (mm)	8.67 ± 2.24	0.98 (0.97-0.99)	4.2	0.35 ± 4.10	0.98	11.39	9.53 ± 2.16	0.98 (0.96-0.99)	4.2	0.31 ± 3.29	0.87	9.15	10.04 ± 2.23	0.98 (0.96-0.99)	3.5	0.33 ± 3.37	0.93	9.36
Tc (ms)	26.25 ± 4.81	0.97 (0.95-0.98)	2.7	0.87 ± 3.33	2.42	9.24	26.19 ± 4.22	0.98 (0.96-0.99)	2.6	0.61 ± 2.34	1.70	6.50	26.30 ± 4.12	0.99 (0.98-0.99)	2.0	0.45 ± 1.72	1.26	4.79
Td (ms)	24.79 ± 1.96	0.93 (0.84-0.96)	2.0	0.53 ± 2.15	1.47	5.96	25.14 ± 1.77	0.94 (0.89-0.96)	2.0	0.45 ± 1.80	1.25	5.00	25.36 ± 1.87	0.94 (0.90-0.97)	2.3	0.46 ± 1.81	1.27	5.04
Tr (ms)	75.64 ± 49.14	0.83 (0.73-0.90)	33.0	23.37 ± 30.89	64.79	85.66	69.34 ± 53.41	0.82 (0.71-0.90)	40.9	28.75 ± 41.46	79.70	114.94	64.96 ± 51.58	0.94 (0.89-0.96)	22	19.68 ± 30.29	46.26	71.21
Ts (ms)	110.95 ± 45.82	0.87 (0.79-0.93)	20.3	17.10 ± 15.41	47.40	42.72	102.17 ± 52.92	0.83 (0.73-0.90)	22.9	26.90 ± 26.32	74.56	72.98	97.92 ± 51.98	0.94 (0.89-0.96)	14.2	16.94 ± 17.29	46.97	47.97

Data are mean ± SD. RF: Rectus Femoris; Dm: displacement; Tc: contraction time; Td: delay time; Tr: half-relaxation time; Ts: sustain time; ms: milliseconds. CV: coefficient of variation; cm: centimeters; ICC: intraclass correlation coefficient; CI: confidence interval; SEM standard error of measurement; MDC: minimum detectable change.

As observed in Table 4, the stimulus length and the electrode size modulated the Tc of the VM with a large effect size (η^2^_p_ = 0.163–0.438). However, none of the conditions modulated the Tc of the RF. In the VM, a smaller electrode size caused a longer Tc response (22.30 vs 21.72 ms; 2.6%, p = 0.001 for 3.2 vs. 5 cm, respectively). In addition, a shorter stimulus length caused a longer Tc between 0.2 and 0.5 ms (22.21 vs 21.83 ms; 1.7%, p = 0.001). However, there were no differences in the Tc between stimuli neither of 0.5 and 1 ms nor between 0.2 and 1 ms.

**Table 4:** Effects of different stimulus length and electrode size on different tensiomography parameters.

	EFFECT	F	Df	P	η^2^
Tc VM	stimulus length*	*6.81*2	*2*	*0.002*	*0*.1*63*
	electrode size *	2*7*.2*44*	1	*0.001*	*0.438*
	stimulus length × electrode size	*1.341*	2	*0*.2*68*	*0.037*
Tc RF	stimulus length	1*.5*2*6*	2	*0.225*	*0.042*
	electrode size	*0.836*	1	*0.367*	*0.0*2*3*
	stimulus length × electrode size	*1.774*	2	*0*.1*77*	*0.048*
Dm VM	stimulus length*	*3.418*	2	*0.038*	*0.089*
	electrode size *	*4.757*	*1*	*0.036*	*0.1*2*0*
	stimulus length × electrode size *	*4.628*	*2*	*0.0*1*3*	*0.117*
Dm RF	stimulus length*	*53.793*	2	*0.001*	*0.606*
	electrode size *	*49.926*	*1*	*0.001*	*0.588*
	stimulus length × electrode size	*0.675*	*2*	*0.513*	*0.019*
Td VM	stimulus length	*2.041*	*2*	*0.138*	*0.055*
	electrode size *	*5.694*	*1*	*0.023*	*0.140*
	stimulus length × electrode size	*0.082*	*2*	*0.922*	*0.002*
Td RF	stimulus length*	*10.865*	*2*	*0.001*	*0.237*
	electrode size *	*17.002*	*1*	*0.001*	*0.327*
	stimulus length × electrode size	*0.386*	*2*	*0.681*	*0.011*

Interactions between stimulus length (0.2, 0.5 and 1 ms), and electrode size (3.2 vs. 5 cm). *p < 0.05.

**Figure 2:**
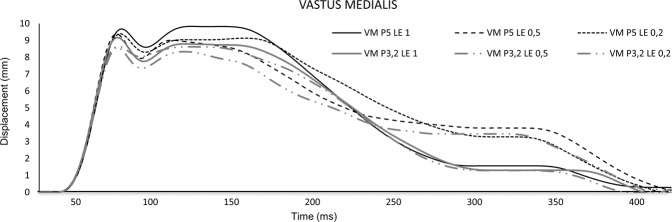
Displacement-time curves of Vastus Medialis of the dominant leg, according to the electrode size (3.2–5 cm) and the stimulus length (0.2, 0.5, and 1 ms).

**Figure 3:**
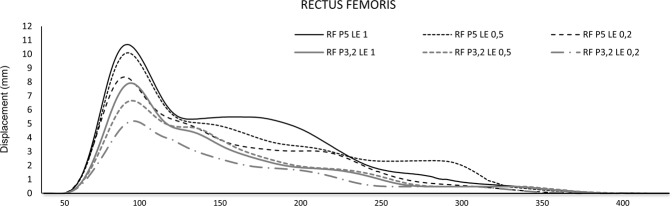
Displacement-time curves of Rectus Femoris of the dominant leg, according to the electrode size (3.2–5 cm) and the stimulus length (0.2, 0.5, and 1 ms).

The Dm of the VM moderately increased (η^2^_p_ = 0.089) as the stimulus length increased, although only significantly between the duration of 0.2 and 0.5 ms (8.41 vs 8.58 mm, 2.0%, p = 0.025) (Fig. 2). The Dm of the RF also increased as the stimulus length increased with a large effect size (η^2^_p_ = 0.606) (Fig. 3). In addition, it also increased 10.4% (p = 0.001) between 0.2 and 0.5 ms duration, 7.0% (p = 0.001) between 0.5 and 1 ms and 18.2% (p = 0.001) between 0.2 and 1 ms. The use of the largest electrode (5 cm) caused a greater Dm than using the smaller one (3.2 cm) in both the VM (2.8%, p = 0.036) and RF (10.8%, p = 0.001). In addition, the interaction stimulus length x electrode size moderately (η^2^_p_ = 0.117) modulated the Dm of the VM, which occurred with all stimulus length (8.18–8.65 mm at 0.2 ms; 8.50–8.67 mm at 0.5 ms; 8.60–8.67 mm at 1 ms; p = 0.013).

The Td of the VM was only significantly affected by the size of the electrode. The use of a larger electrode (5 cm) caused a lower Td (22.28 vs 22.11 ms, 0.7%, p = 0.023). However, the Td of the RF increased as the stimulus length increases with a large effect size (η^2^_p_ = 0.237). It increased by 1.4% (p = 0.006) between 0.2 and 0.5 ms duration, 0.7% (p = 0.036) between 0.5 and 1 ms and 2.1% (p = 0.001) between 0.2 and 1 ms. In addition, the electrode size also modulated the Td of the RF with a large effect size (η^2^_p_ = 0.327), so that the use of the largest electrode (5 cm) caused longer Td (1.4%, p = 0.001).

## Discussion

Summarizing, the results of this study show that the stimulus length (duration of the TNS pulse) applied to the RF and VM muscles and the size of the electrode significantly influence the muscle’s contractile properties as measured by a single-twitch TMG technique. The main finding is that Dm increases significantly both, as the duration of the TNS increases and with the use of a larger electrode (5 cm) in both muscles. However, Tc and Td are less affected by both conditions and not entirely clear.

Our results are consistent with previous reviews on the topic showing that Tr parameter should not be used in research or clinical environments due to their insufficient absolute reliability and high measurement error^2,16^. Similarly, Ts parameter should not be used in these areas since it has shown a CV higher than 10%^17^. On the contrary, Dm, Tc and Td parameters have shown a high relative reliability in all muscles and situations evaluated, which is in line with the findings of Lohr *et al*.^35^ but also an absolute reliability through a low CV, %SEM and %MDC, as opposed to the results of Lohr *et al*.^35^ which indicates a discordant absolute reliability in the Dm and moderate positive in the Tc. Hence, the effect of stimulus length and electrode size on Tr and Ts was not examined, since these parameters did not meet the reliability requirements.

The electrical energy available to stimulate the muscle is influenced by the duration of the applied electrical impulse as shown by the equation^20^: $(E=V\,x\,I\,x\,\Delta t),\,$where E is the electrical energy, V are volts, I equals current and $\Delta \,$t is the duration of the TNS pulse. It appears that, at stimulus length below 1 ms, there is insufficient electrical energy available to maximally stimulate all motor units^36^. Moreover, given that smaller motor units are easier to stimulate than larger motor units^37,38^, shorter stimulus length appear to favor type I slow twitch muscle fibers over type II fast twitch muscle fibers, which are more difficult to activate^36^. Our results match those found by McAndrew *et al*.^14^, who reported that stimulus length above 0.3 ms provide both a maximal lateral displacement of the muscle’s belly and stable measures of its contractile properties. Although the previous authors used a laser-based MMG technique, their results can be extrapolated to ours since it has been demonstrated that both laser- and CDS shows good-to-excellent reliability for the assessment of muscle contractile properties with no significant differences between them^15^. In fact, it should be noted that there are data showing that the radial muscle displacement (Dm) increases linearly with muscle torque up to 68% of maximal voluntary contraction^6^. This fact may suggest that, maintaining linearity, using shorter pulse duration (0.2 ms) could elicit low torque production, affecting to the wave-form and then to all the TMG parameters.

To our knowledge, to date there has been no study that has analysed the effects of the electrodes size for muscle electrical stimulation measured by TMG. However, it has been previously analysed in transcutaneous electrical nerve stimulation (TENS)^39^. This author used 4 types of electrodes: 3 × 3, 6 × 6 9 × 9 and 5 × 16.2 cm on the quadriceps with the objective of examining the effects of electrode size at neural and motor level, reaching the conclusion that the larger the electrode, the greater the participation of motor units. Our results seem to be in agreement with those obtained by Alon^39^ with respect to the electrode size, since both VM and RF increased their Dm with the largest electrode. A reasonable explanation is that a larger electrode is able to recruit more motor units on the transverse axis and therefore greater amount of muscle mass displacement directly influences an increase in Dm. However, this hypothesis will still have to be proven.

On the other hand, the overall %SEM and %MDC results of both muscles seem to point out, in a general way, that increasing electrode size and pulse duration could increase the data accuracy and minimize the measurement error, although it would be necessary more research with other muscle groups to verify this point. *A priori*, to minimize measurement errors with TMG parameters, it should be recommended standardizing TMG protocols to develop a TMG standard operating procedure such that experimental studies may be comparable. In order to do this, based on the present findings and previous research, it is recommended: (1) to take into account the correct stimulus length (1 ms) to maximize Dm; (2) to use electrodes of 5 cm; (3) to use specific guides for each muscle of inter-electrode distance^18,19^; and (4) additional recommendations for a correct measurement protocol are described in the review of Macgregor *et al*.^2^. In this line, Lohr *et al*.^35^ have indicated that it is necessary to have high methodological standards for conduct and reporting TMG studies, and these recommendations could be helpful for this purpose.

## Limitations, strengths and practical applications

Finally, it is important pointing that, the maximal TNS voltage required to produce peak lateral displacement of a muscle’s belly is unique to each muscle and is determined as the voltage that first produces an undistorted parabolic MMG waveform of maximal amplitude^14^. In this regard, we used a unique intensity (100 mA) in our study to be able to homogenize and compare subjects and muscles with each other. This was done because the current and well accepted ramp protocol^2,8^, used to decide individual intensity would have made it difficult to compare results between subjects’ muscles. This could have been solved by normalizing the values of each parameter of each subject/muscle to, for example, a maximum voluntary contraction. However, we did not have the necessary tools for this purpose, what is a limitation of this study. In addition, a duration of TNS above 1 ms could also have been used to try to stimulate larger motor units, but the TMG software did not allow this when the experiment was performed. Furthermore, it has only been evaluated two muscles, both of the lower limb, which may be another limitation of this study. On the other hand, the main strength of the present study is the sample size and the use of several set configurations for both electrode size and stimulus pulse duration, and as well as that the same evaluator with more of 7 years’ experience took all measurements. In terms of practical applications, TMG researchers and practitioners can base their measurement protocol on the findings of this study. Our data indicates that a stimulation pulse duration of 1 ms together with the election of a 5 × 5 cm electrode size is necessary to reach a reliable and reproducible assessment of both RF and VM muscles contractile properties. Conversely, the use of a smaller electrode or a stimulus length of less than 1 ms would be a risk to guarantee the reproducibility of the measurement taken with TMG.

## Conclusions

The duration of the stimulus length applied to the RF and VM muscles and electrode size significantly influence the muscle’s contractile properties as measured by a single-twitch TMG technique. In fact, the Dm increases substantially as the duration of the TNS increases and with the use of the larger electrode (5 cm) in both muscles. However, Tc and Td are less affected by both conditions and not entirely clear. Therefore this study indicates that a stimulus pulse duration of 1 ms together with the election of a 5 × 5 cm electrode size is necessary to reach a reliable and reproducible assessment of both rectus and vastus medialis muscles contractile properties.

## Data Availability

The TMG recordings utilized in the current study will be uploaded to an open access server and will be available to anyone who would like to re-analyse them.
